# Survival analysis and sentinel lymph node status in thin cutaneous melanoma: A multicenter observational study

**DOI:** 10.1002/cam4.2358

**Published:** 2019-06-18

**Authors:** Antonio Tejera‐Vaquerizo, Simone Ribero, Susana Puig, Aram Boada, Sabela Paradela, David Moreno‐Ramírez, Javier Cañueto, Blanca de Unamuno, Ana Brinca, Miguel A. Descalzo‐Gallego, Simona Osella‐Abate, Paola Cassoni, Cristina Carrera, Sergi Vidal‐Sicart, Antoni Bennássar, Ramón Rull, Llucìa Alos, Celia Requena, Isidro Bolumar, Víctor Traves, Ángel Pla, A. Fernández‐Orland, Ane Jaka, María T. Fernández‐Figueres, Josep M. Hilari, Pol Giménez‐Xavier, Ricardo Vieira, Rafael Botella‐Estrada, Concepción Román‐Curto, Lara Ferrándiz, Nicolás Iglesias‐Pena, Carlos Ferrándiz, Josep Malvehy, Pietro Quaglino, Eduardo Nagore, on behalf of SENTIMEL group

**Affiliations:** ^1^ Dermatology Department Instituto Dermatológico GlobalDerm Palma del Río, Córdoba Spain; ^2^ Medical Sciences Department, Section of Dermatology University of Turin Turin Italy; ^3^ Melanoma Unit, Dermatology Department, Hospital Clinic Universitat de Barcelona, Institut d'investigacions biomèdiques August Pi i Sunyer (IDIBAPS) Barcelona Spain; ^4^ Centro de Investigación Biomédica en Red (CIBER) de Enfermedades Raras Barcelona Spain; ^5^ Departamento de Dermatología Hospital Universitari Germans Trial i Pujol Badalona Spain; ^6^ Departamento de Dermatología Hospital Universitario de la Coruña La Coruña Spain; ^7^ Melanoma Unit, Medical‐&‐Surgical Dermatology Department Hospital Universitario Virgen Macarena Sevilla Spain; ^8^ Servicio de Dermatología Complejo Asistencial Universitario de Salamanca Salamanca Spain; ^9^ Instituto de Investigación Biomédica de Salamanca Complejo Asistencial Universitario de Salamanca Salamanca Spain; ^10^ Departamento de Dermatología Hospital Universitario La Fe Valencia Spain; ^11^ Department of Dermatology University Hospital of Coimbra Coimbra Portugal; ^12^ Unidad de Investigación Academia Española de Dermatología Fundación Piel Sana Madrid Spain; ^13^ Medical Sciences Department, Section of Surgical Pathology University of Turin Turin Italy; ^14^ Nuclear Medicine Department Hospital Clinic Barcelona, Universitat de Barcelona, Institut d'investigacions biomèdiques August Pi i Sunyer (IDIBAPS) Barcelona Spain; ^15^ Surgery Department Hospital Clinic Barcelona Spain; ^16^ Pathology Department, Hospital Clinic Universidad de Barcelona Barcelona Spain; ^17^ Dermatology Department Instituto Valenciano de Oncología Valencia Spain; ^18^ Surgery Department Instituto Valenciano de Oncología Valencia Spain; ^19^ Pathology Department Instituto Valenciano de Oncología Valencia Spain; ^20^ Otorhinolaringology Department Instituto Valenciano de Oncología Valencia Spain; ^21^ Pathology Department Hospital Universitari Germans Trial i Pujol Badalona Spain

**Keywords:** age factors, Cox proportional hazards regression analysis, melanoma, mitotic index, multiple imputation, neoplasia staging, prognostic factors, sentinel lymph node biopsy, survival

## Abstract

Mitotic rate is no longer considered a staging criterion for thin melanoma in the 8th edition of the American Joint Committee on Cancer Staging Manual. The aim of this observational study was to identify prognostic factors for thin melanoma and predictors and prognostic significance of sentinel lymph node (SLN) involvement in a large multicenter cohort of patients with melanoma from nine tertiary care hospitals. A total of 4249 consecutive patients with thin melanoma diagnosed from January 1, 1998 to December 31, 2016 were included. The main outcomes were disease‐free interval and melanoma‐specific survival for the overall population and predictors of SLN metastasis (*n* = 1083). Associations between survival and SLN status and different clinical and pathologic variables (sex, age, tumor location, mitosis, ulceration, regression, lymphovascular invasion, histologic subtype, Clark level, and Breslow thickness) were analyzed by Cox proportional hazards regression and logistic regression. SLN status was the most important prognostic factor for melanoma‐specific survival (hazard ratio, 13.8; 95% CI, 6.1‐31.2; *P* < 0.001), followed by sex, ulceration, and Clark level for patients who underwent SLNB. A mitotic rate of >2 mitoses/mm^2^ was the only factor associated with a positive SLN biopsy (odds ratio, 2.9; 95% CI, 1.22‐7; *P* = 0.01. SLN status is the most important prognostic factor in thin melanoma. A high mitotic rate is associated with metastatic SLN involvement. SLN biopsy should be discussed and recommended in patients with thin melanoma and a high mitotic rate.

## 1 INTRODUCTION

Thin melanomas measuring 1 mm or less account for approximately 50% of all melanomas diagnosed in our area.^1^ Although thin melanomas have a good prognosis, the absolute number of deaths is very high due to the sheer number of cases.^2^

Before the 8th edition of the American Joint Committee on Cancer (AJCC) Cancer Staging Manual,^3^ most clinical guidelines recommended sentinel lymph node biopsy (SLNB) for patients with thin melanoma and additional risk factors, such as tumor thickness ≥0.75 mm, ulceration, mitosis, and a high Clark level.^4^ The decision to perform SLNB in patients with melanoma ≤1 mm should be made by weighing the likelihood of detecting SLN metastasis, established as an expected rate of at least 5%,^5^ against the potential complications, not to mention cost, of the procedure.^6^ A recent systematic review and meta‐analysis established that the presence of at least one mitosis was the most important predictor of SLN metastasis with respect to adjusted variables in patients with thin melanoma.^7^ However, in the new edition of the AJCC Cancer Staging Manual,^3^ mitotic rate is no longer considered a staging criterion for thin melanoma, although its prognostic value is still clearly recognized. The only two updated clinical practice guidelines—version 1.2018 of the National Comprehensive Cancer Network (NCCN) guidelines (version 1.2018)^8^ and the American Society of Clinical Oncology‐Society of Surgical Oncology (ASCO‐SSO) guideline^9^—recommend discussing and considering SLNB in patients with T1b melanoma (ulcerated tumors or tumors with a Breslow thickness ≥0.8 mm). The NCCN guidelines additionally recommend considering SLNB in patients with T1a melanoma (tumors <0.8 mm without ulceration) when ≥2 mitoses/mm^2^ are observed, especially in young patients or patients with lymphovascular invasion.

The main objective of this study was to identify prognostic factors for thin melanoma and predictive factors associated with SLN involvement in a large multicenter cohort of patients with melanoma.

## 2 MATERIALS AND METHODS

We designed a multicenter observational study based on the Strengthening the Reporting of Observational Studies in Epidemiology guidelines.^10^ Patients were recruited in nine hospitals that formed part of the Sentinel Lymph Node Study Group in Melanoma (SENTIMEL). Patients with melanoma were selected from the databases of seven Spanish hospitals (Instituto Valenciano de Oncología [IVO] in Valencia, Hospital Universitario de Salamanca [HUS] in Salamanca, Hospital La Fe [HLF] in Valencia, Hospital Virgen Macarena [HVM] in Seville, Hospital de la Coruña [HC] in A Coruña, Hospital German Trials i Pujol [HGT] in Badalona, and Hospital Clìnic [HC] in Barcelona) and two hospitals outside Spain (Hopital de Coimbra [HCo] in Portugal and Hospital de Turin [HT] in Italy). All the hospitals are tertiary care hospitals and reference centers for melanoma. Patients registered in any of the databases up to December 31, 2016 were included in the study. Patients diagnosed after January 1, 1998 were included. Those treated before this date were intentionally omitted, as most of the hospitals did not start performing SLNB in patients considered at risk for regional lymphatic metastasis until 1998.^3^ All the databases complied with the relevant laws. The study was approved by the institutional review board of Hospital Universitario Reina Sofía in Córdoba (Spain) which acted as a reference committee.

### 2.1 Inclusion criteria and case definition

All patients ≥18 years old with a solitary localized melanoma, without evidence of metastasis at diagnosis with a thickness of 1 mm or less were analyzed, regardless of whether or not they had undergone SLNB. They had been included consecutively and prospectively in the relevant databases. SLNB was performed in a similar way in all centers and used any combination of vital blue dye, radioactive dye, radioactive colloid and preoperative lymphoscintigraphy for location SLN. The surgical management of primary melanoma was performed with a 1 cm wide excision, as recommended by most guidelines.

Pathologic examination of the sentinel lymph nodes at each hospital was performed using standard procedures that have previously been described.^11^ The HCUB has been applying the Minitub protocol (EORTC 1208: Minitub registration study) since 2011.

### 2.2 Independent variables

Drawing on the literature and the available evidence, the following clinical and pathologic variables were chosen as independent variables:

Clinical characteristics: sex, age (continuous variable classified as <40, 40‐65, and >65 years), and anatomic location (head/neck, trunk, upper extremities, or lower extremities).

Histologic characteristics: Breslow thickness, mitotic rate, ulceration, regression, lymphovascular invasion, Clark level, and histologic subtype. For thickness, patients were reclassified according to the new AJCC staging criteria (<0.8 mm vs ≥0.8 mm). Thickness was rounded to the nearest decimal point (0.1 mm), as recommended by the AJCC.^3^ Mitotic rate (number of mitoses/mm^2^) was evaluated using the hot spot method, which consists of identifying the area of the dermis with the highest number of mitotic figures and counting the mitoses in adjacent fields until an area of 1 mm^2^ is reached. The previously established cutoffs of 0, 1, 2, and >2 mitoses/mm^2^ were used.^12^ Ulceration, regression, and lymphovascular invasion were evaluated as presence or absence and Clark level was divided into levels II‐III vs IV‐V.

The type of recurrence was recorded (locoregional, regional or distant recurrence).

### 2.3 Outcomes

The primary outcome measures for the overall population were disease‐free interval (DFI) and melanoma‐specific survival (MSS). Survival was measured from time of primary tumor excision to time of local, regional lymphatic, or distant recurrence or death due to melanoma (respective events for DFI and MSS), or to time of last follow‐up or death due to a cause other than melanoma (whichever happened first). National death records were used by some hospitals to evaluate the vital status in patients.

### 2.4 Statistical analysis

Difference between SLN status and variables was investigated with chi‐square test. Survival curves were built using the Kaplan‐Meier method and compared using the log rank test. Cox proportional hazards regression analysis was used for the multivariate analysis including and the assumption of proportionality was evaluated graphically using a log‐log graph. Missing values were imputed using multiple imputation, for which 10 full datasets were created. The estimates were combined using Rubin's rules.^13^ Before running the model, SPSS's missing value add‐on module was used to check whether the missing data were missing at random. The variables that have been imputed were age, sex, anatomic location, mitotic rate, ulceration histologic, subtype, lymphovascular invasion, regression and Clark level, with different levels of missingness. And variables without missing values were used to impute and recreate the scenario of values under missing at random assumption (MAR).

The dependent variable for patients who underwent SLNB was a positive result. Binomial logistic regression analysis was used to evaluate the effects of the independent variables on the likelihood of SLN positivity, using variables potentially associated with SLN status in the univariate model (*P* < 0.2) for the multivariate model with a backward stepwise elimination and multivariate analysis of Cox proportional hazards models were performed after multiple imputation and were adjusted for all the study variables: age, sex, anatomic location, Breslow thickness, ulceration, mitotic rate, histologic subtype, Clark level, lymphovascular invasion, and regression. The variable *hospital* was not included in the binomial logistic regression model to avoid oversaturation.

Statistical significance was set at a P value of less than 0.05. All statistical analyses were performed in SSPS 20.0 (Illinois, Inc; USA).

## 3 RESULTS

We analyzed data for 4249 patients with thin melanoma (≤1 mm); of this entire cohort, 1897 patients were men (44.6%) and 2352 were women (55.4%). The median age was 57.8 years (interquartile range, 40‐66 years). The most common age for diagnosis was 41‐65 years (48.1%) and the most common anatomic location was the trunk (42.4%). Absence of mitosis was the most common finding (67%) and superficial spreading melanoma was the most common histologic subtype. A tumor thickness of <0.8 mm was reported for 71% of patients. The characteristics of the population are summarized in Table 1.

**Table 1:** Characteristics of 4249 patients with thin melanoma according to the 8th edition of the American Joint Committee on Cancer Staging Manual

Characteristic	No.	%
Sex		
Male	1897	44.6
Female	2352	55.4
Age of diagnosis		
Median	57.8	
p75‐p25	40‐66	
≤40 y old	1120	26.5
41‐65 y old	2034	48.1
>65 y old	1071	25.3
Anatomic location (missing = 133; 3.1%)		
Head and neck	494	12
Trunk	1746	42.4
Upper extremities	1261	30.6
Lower extremities	615	14.9
Breslow thickness		
Median	0.6	
p75‐p25	0.4‐0.8	
<0.8	3018	71
0.8‐1.0	1231	29
Ulceration (missing = 495; 11.6%)		
Absent	3613	96.2
Present	141	3.8
Mitotic rate (missing = 1752; 41.2%)		
0 mitoses/mm^2^	1674	67
1 mitosis/mm^2^	514	20.6
2 mitoses/mm^2^	176	7
>2 mitoses/mm^2^	133	5.3
Histologic subtypes (missing = 64; 1.5%)		
LMM	346	8.3
SSM	3487	83.3
NM	89	2.1
ALM	94	2.2
Other	169	4
Clark level (missing = 973; 22.9%)		
II‐III	2838	86.6
IV‐V	438	13.4
Lymphovascular invasion (missing = 1887; 44.4%)		
Absent	2287	99.3
Present	15	0.4
Regression (missing = 848; 20%)		
Absent	2175	64
Present	1226	36
Hospital		
1	130	3.1
2	698	16.4
3	984	23.2
4	1210	28.5
5	505	11.9
6	80	1.9
7	284	6.7
8	247	5.8
9	109	2.6

Notes

Hospital: 1. Hospital Universitario de Salamanca (Salamanca, Spain); 2. Instituto Valenciano de Oncología (Valencia, Spain); 3. Hospital Clìnic (Barcelona, Spain); 4. Hospital de Turin (Turin, Italy); 5. Hospital German Trials i Pujol (Badalona, Spain); 6. Hospital de Coimbra (Coimbra, Portugal); 7. Hospital Universitario A Coruña (A Coruña, Spain); 8. Hospital Virgen Macarena (Seville, Spain); 9. Hospital La Fe (Valencia, Spain).

Abbreviations: ALM, acral lentiginous melanoma; LMM, lentigo maligna melanoma; NM, nodular melanoma; SSM, superficial spreading melanoma.

After a median follow‐up of 68 months, 158 patients (3.7%) experienced disease recurrence (locoregional 22.9%, regional 34.6%, and distant 42.5%) and 77 (1.8%) died of melanoma (uncensored in the survival analysis).

For the overall population (*n* = 4249), sex, Breslow thickness, ulceration, Clark level, and histologic subtype were all significantly associated with DFI and MMS (Table 2). On analyzing SNLB patients only (*n* = 1083), SLN status was the most important prognostic factor for both DFI (hazard ratio 7.4; 95% CI, 4.1‐13.5) (*P* < 0.001) and MSS (hazard ratio, 12.8; 95% CI 5.4‐30.2) (*P* < 0.001). Additional independent predictors of DFI and MSS were sex, age, Clark level, and ulceration (Table 2).

**Table 2:** Multivariate Cox regression model for disease‐free interval and melanoma‐specific survival in the entire cohort and in patients who underwent sentinel lymph node biopsy

Variable	Hazard ratio	95% CI	*P* value
Entire cohort (*n* = 4249)			
Disease‐free interval			
Sex (female vs male)	0.7	0.5‐0.8	0.006
Ulceration (presence vs absence)	2.4	1.3‐4.3	0.006
Others vs LMM	0.3	0.2‐0.9	0.031
≥0.8 mm vs <0.8 mm Breslow thickness	1.6	1.1‐3	0.01
Clark level (IV‐V vs II‐III)	3.1	2‐4.8	<0.001
Melanoma‐specific survival			
Sex (female vs male)	0.4	0.2‐0.7	<0.001
Ulceration (presence vs absence)	2.9	1.4‐6.1	0.005
ALM vs LMM	15.1	2.3‐98.6	0.004
≥0.8 mm vs <0.8 mm Breslow	1.9	1.1‐3.2	0.021
Clark level (IV‐V vs II‐III)	3	1.6‐5.7	0.001
Patients underwent SLNB (*n* = 1090)			
Disease‐free interval			
Sex (female vs male)	0.5	0.3‐0.9	0.027
Age >5 vs <40	2.5	1.1‐5.7	0.028
Ulceration (presence vs absence)	3	1.5‐5.9	0.002
Clark level (IV‐V vs II‐III)	2.8	1.6‐4.9	<0.001
SLNB (positive vs negative)	7.4	4.1‐13.5	<0.001
Melanoma‐specific survival			
Sex (female vs male)	0.3	0.1‐0.6	0.004
Age >65 vs <40	3.5	1‐12.1	0.044
Ulceration (presence vs absence)	4.3	1.7‐10.4	0.001
Clark level (IV‐V vs II‐III)	2.6	1.1‐6.1	0.022
SLNB (positive vs negative)	12.8	5.4‐30.2	<0.001

Abbreviations: ALM, acral lentiginous melanoma; LMM, lentigo maligna melanoma; NM, nodular melanoma; SSM, superficial spreading melanoma; SLNB: Sentinel lymph node biopsy.

**Figure 1:**
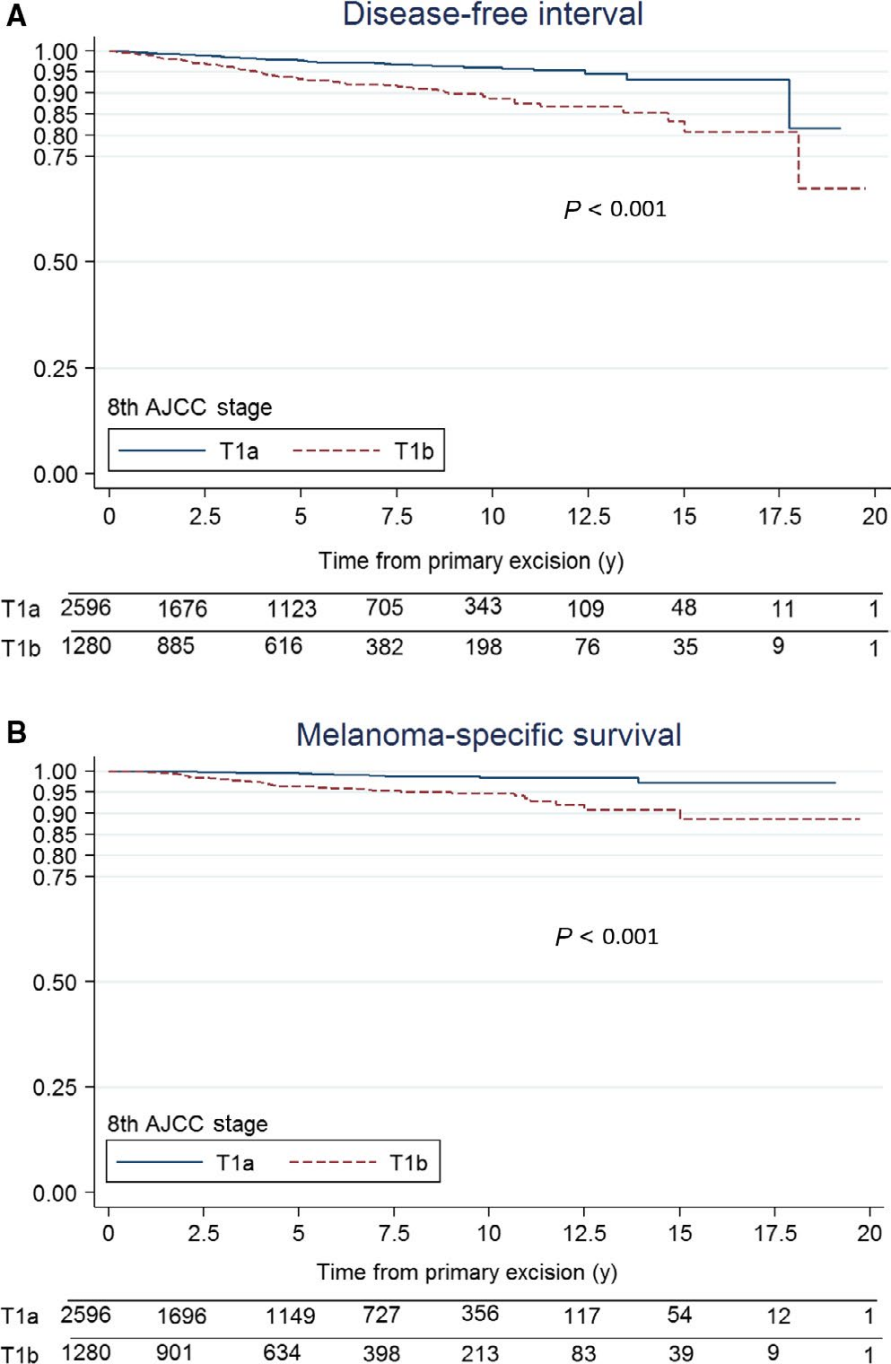
Kaplan‐Meier disease‐specific survival curves for patients with thin melanoma according to the staging criteria of the 8th edition of the American Joint Committee on Cancer Staging Manual, T1a vs T1b

The cumulative estimated survival rates for T1a melanoma patients at 5, 10, and 15 years, respectively, were 97.6%, 96.1%, 93.2% for DFI, and 99.5%, 98.5%, and 97.2% for MSS (Figure 1A and 1B) (*P* < 0.001). The corresponding rates for patients with T1b melanoma were 93.3%, 88.5%, and 80.8% for DFI and 96.3%, 94.8%, and 88.6% for MSS (Figure 1A and 1B) (*P* < 0.001).

The characteristics of the patients who underwent SLNB are summarized by SLN status in Table 1. Of the 1083 patients with thin melanoma who underwent SLNB, 73 (6.7%) had a positive result. The median of positive SLNB patients was higher than negative SLNB (0.9 vs 0.8 mm). Only Breslow thickness (odd ratio [OR] 1.7 [95% CI, 1‐2.92]; *P* = 0.05) and mitotic rate (>2 mitoses/mm^2^) (OR, 3.4 [95% CI, 1.5‐7.7]; *P* = 0.003) were associated with a positive status in the univariate analysis. In the multivariate analysis, only mitotic rate retained its prognostic significance (OR, 2.9 [95% CI, 1.22‐7]; *P* = 0.01) (Table 3).

**Table 3:** Univariate and multivariate analysis of predictive factors for positive sentinel lymph node biopsy in 1090 patients with thin melanoma

Independent variable	Univariate analysis		Multivariate analysis	
	*P* value	Odds ratio (95% CI)	*P* value	Odds ratio (95% CI)
Sex				
Female		1		—
Male	0.21	0.74 (0.52‐1.21)	—	—
Age, y				
<40		1		
40‐65	0.25	0.72 (0.41‐1.26)	—	—
>65	0.78	0.91 (0.47‐1.75)	—	—
Anatomic location				
Head/neck		1		—
Trunk	0.89	1.06 (0.43‐2.59)	—	—
Upper extremities	0.57	0.75 (0.29‐1.96)	—	—
Lower extremities	0.73	0.84 (0.30‐2.32)	—	—
Breslow thickness				
<0.8 mm		1		
0.8‐1 mm	0.05	1.7 (1‐2.92)	—	—
Ulceration				
Present	0.6	1.22 (0.61‐1.63)	—	—
Absent		1		
Mitosis rate				
0 mitoses/mm^2^		1		1
1 mitosis/mm^2^	0.5	1.3 (0.6‐2.9)	0.5	1.3 (0.6‐2.9)
2 mitoses/mm^2^	0.06	2.23 (0.97‐5.14)	0.08	2.1 (0.9‐4.95)
>2 mitoses/mm^2^	0.003	3.42 (1.52‐7.73)	0.01	2.9 (1.22‐7)
Regression				
Present		1		—
Absent	0.39	1.27 (0.73‐2.19)	—	—
Clark level				
II‐III		1		—
IV‐V	0.18	1.41 (0.84‐2.37)	—	—
Histologic subtype				
LMM		1		—
SSM	0.61	1.44 (0.31‐6.13)	—	—
NM	0.34	2.22 (0.42‐11.7)	—	—
Others	0.47	0.48 (0.06‐3.46)	—	—
Lymphovascular invasion				
Present	0.71	1.44 (0.19‐10.9)	—	—
Absent		1		

The frequency of positive SLNB results for each combination of mitotic rate and clinical stage (T1a vs T1b) was evaluated according to the different recommendations and criteria described in the NCCN and ASCO‐SSO guidelines (Table 4). Patients with T1a melanoma had an SLN positivity rate of 3.4% and this rose to 20% in those with ≥2 mitosis/mm^2^. The overall SLN positivity rate in patients with T1b melanoma was 8%, but this fell to 3.6% in the subgroup of patients with 0 mitosis/mm^2^. Based on the NCCN recommendations for stage T1a thin melanoma with ≥2 mitosis/mm^2^, the SLN positivity rate was 7.8%. None of the 17 patients in the younger age group (<40 years) had a positive SLNB. We were unable to evaluate the frequency of positive SLNB results in patients with lymphovascular invasion, as there were just three cases.

**Table 4:** Association between SLN status and different tumor staging criteria for thin melanoma within the framework of current guideline recommendations for SLN biopsy

Scenario	All patients	SLN positivity *n* (%)	NCCN guidelines	ASCO‐SSO guideline	Present study
T1a (NCCN)					
≥2 mitoses/mm^2^	51	4 (7.8)	DC	NR	DC
Lymphovascular invasion	3	0 (0)	DC	NR	**
≥2 mitoses/mm^2 ^+ young age^a^	17	0 (0)	DC	NR	NR
T1a (present study)					
All	358	12 (3.4)	NR	NR	NR
0 mitoses/mm^2^	84	1 (1.2)	NR	NR	NR
1 mitosis/mm^2^	121	3 (2.5)	NR	NR	NR
2 mitoses/mm^2^	36	1 (2.8)	DC	NR	NR
>2 mitoses/mm^2^	15	3 (20)	DC	NR	DC
T1b (present study)					
All	713	57 (8)	DC	DC	DC
0 mitoses/mm^2^	168	6 (3.6)	DC	DC	NR
1 mitosis/mm^2^	164	11 (6.7)	DC	DC	DC
2 mitoses/mm^2^	80	11 (13.8)	DC	DC	DC
>2 mitoses/mm^2^	68	9 (13.2)	DC	DC	DC

Abbreviations: DC, discuss and consider; NR: not recommended; SLN, sentinel lymph node.

a <45 years old.

** Insufficient number.

**Figure 2:**
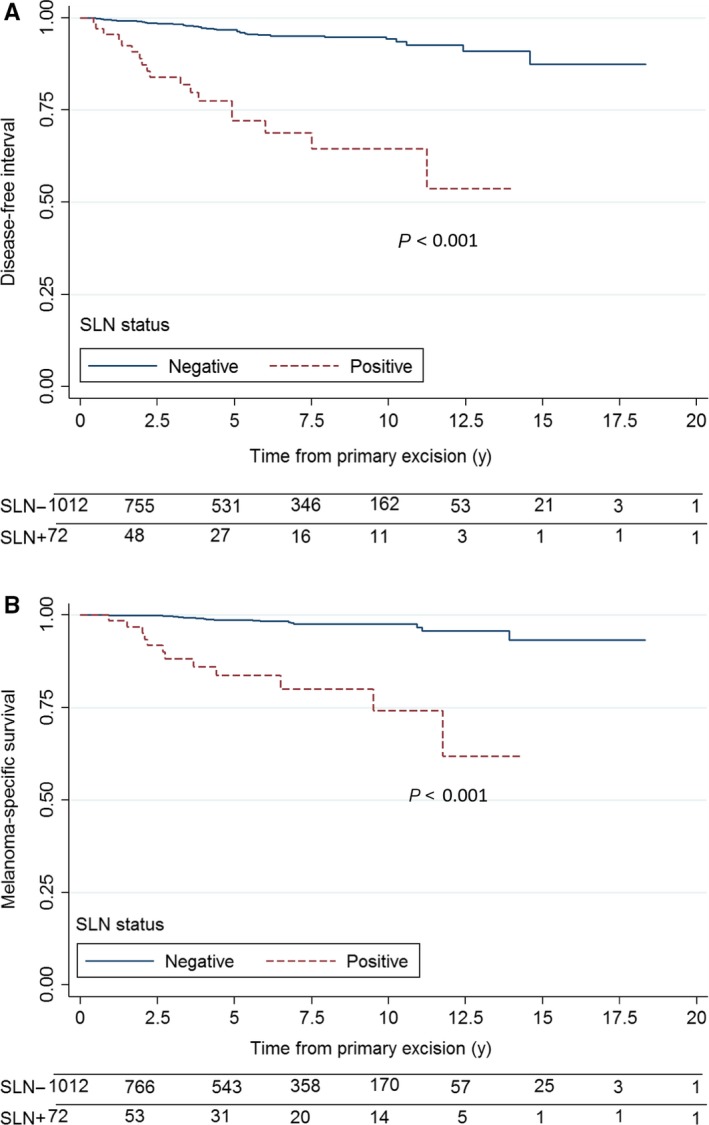
Kaplan‐Meier survival curves for patients with thin melanoma according to sentinel lymph node status: (A) Disease‐free interval; (B) Melanoma‐specific survival

Over a median follow‐up of 64 months for patients in the SLNB subgroup, 58 patients experienced disease recurrence (locoregional 26.3%, regional 19.3%, and distant 54.5%) and 30 died of melanoma. DFI at 5 and 10 years was, respectively, 96.5% and 94.1% for SLN‐negative patients and 72.2% and 64.5% for SLN‐positive patients (*P* < 0.001)(Figure 2A). Five‐and 10‐year MSS, by contrast, was 98.7% and 97.6% for SLN‐negative patients and 83.8% and 74.3% for SLN‐positive patients (*P* < 0.001) (Figure 2B).

The number of false negative patients was nine. The false negative rate was 10.8%. This was calculated by dividing the number of false negatives by the sum of false negatives plus true positives following van Akkooi's method.^14^

## 4 DISCUSSION

This study shows that SLN status is the most important prognostic factor in thin melanoma. Coinciding with the revised AJCC staging system,^3^ the most important factors for MSS, not counting SLN status, were Breslow thickness and ulceration, followed by sex, Clark level, and acral lentiginous melanoma.

It is noteworthy that 5‐year MSS of positive SLNB patient is worse in our study with respect to other similar studies_._ 15, 16 It is probably due to a difference in the selection of patients who underwent the SLNB.

Female sex confers a survival benefit in thin melanoma. Although this benefit is widely recognized,^17^, ^18^ no logical explanation has yet been found.

The findings of our study support a prognostic role for Clark level in thin melanoma. Clark level, defined by Wallace Clark,^19^ was considered a prognostic factor in thin melanoma in earlier editions of the AJCC Cancer Staging Manual, but it was replaced by mitogenicity in the 6th edition.^20^ Clark level is less reproducible than tumor thickness for pathologists as its interpretation is limited by differences between the papillary and reticular dermis in patients with solar elastosis or in certain areas, such as the scalp, acral sites, and mucosal membranes.

Acral lentiginous melanoma is a subtype of melanoma defined by the World Health Organization.^21^ The Clark classification of melanomas, which is based on different epidemiological and genetic features, is not free of criticism due to the overlapping of the histopathologic criteria on which it is based.^22^

Several reasons for the worse prognosis associated with acral lentiginous melanoma have been postulated, including differences in mutations and amplifications, such as a lower incidence of *BRAF*/*NRAS* mutations,^20^ a higher incidence of *KIT* mutations or amplifications,^23^ and amplification of the *TERT* gene^24^ in melanomas classified using the Clark system.

Although SLN status has been identified as the most important prognostic factor in thin melanoma,^16^, ^25^ there is no consensus on which patients should be offered SLNB. The updated ASCO‐SSO guideline does not recommend SLNB for T1a melanomas.^9^ Version 1.2018 of the NCCN melanoma guidelines, however, which incorporate the AJCC's latest modifications to the staging criteria for thin melanoma,^8^ recommend the procedure if the risk of a positive SLN is more than 5%. In the specific case of thin melanoma, it recommends considering SLNB in patients with “T1a lesions <0.8 mm with other adverse features (eg. very high mitotic index ≥2/mm^2^ [particularly in the setting of young age], lymphovascular invasion, or a combination of these factors).” This recommendation, however, is not supported by clinical studies.

Our findings support the indication for SLNB in patients with T1a melanoma and a high mitotic rate (>2/mm^2^). We found no significant associations with patient age. Lymphovascular invasion was not included in the regression model as it was only identified in 15 patients and none of them had SLN metastasis.

Another noteworthy finding in our study was the low rate of SLN positivity (just 3.6%) in patients with T1b melanoma without mitosis. In a recent study of 203 cases of thin melanoma, a similarly low rate was reported for patients with T1b melanoma without mitoses or with a low mitotic rate.^12^ Bartlett et al,^26^ in a study of 781 patients with thin melanoma who underwent SLNB, also found absence of mitotic activity to be associated with a low probability of SLN metastasis, even in patients with thin melanomas >0.75 mm. Although the ASCO‐SSO and NCCN clinical practice guidelines recommend SLNB for patients with T1b melanoma, perhaps the option of not performing the procedure should be contemplated in patients without mitoses.

Previous studies have not found significant associations between mitotic rate and SLN status in thin melanoma,^27^, ^28^, ^29^ but this is probably because they analyzed small samples or used different cutoff points.^16^ Han et al^15^ is one of the most numerous studies, did not find any factor related to at least 5% of possibility to have a positive SLN in melanomas <0.75 mm in dept. In a recent study of thin melanomas from the US National Cancer Database, Wheless et al^30^ observed a positive linear relationship between mitotic rate and increased odds of SLN involvement. A greater likelihood of SLN metastasis in the presence of dermal mitoses in thin melanoma has also been reported elsewhere.^31^, ^32^ Unlike us, however, the authors did not quantify the number of mitoses.

Our study has some limitations, including its retrospective design and the fact that data were missing for some variables. Information on mitotic rate, for example, was missing for 41.2% of patients. On analyzing the differences between patients with and without data on mitotic rate, we found no differences for Breslow thickness or ulceration (data not shown). Another possible limitation of our study is that it was performed in southern Europe, where there is a low incidence of melanoma compared with other areas of the continent. The SLN positivity rate for patients with thin melanoma in our study was 6.7%, but on counting the false negatives, it was 7.5%, which is higher than in other series.^26^ It can be assumed, however, that this higher rate did not affect the aim of the study. Our study also has important strengths, such as its multicenter design and the large number of patients analyzed. Finally, the adequacy of the follow‐up period is assured by the similarities observed between the survival rates in our study and those reported by the AJCC for thin melanoma.

## 5 CONCLUSIONS

SLN involvement is the most important prognostic factor among patients undergoing SLNB in thin melanoma. High mitotic rate is associated with SLN metastasis but probably has no impact on survival in patients with thin melanoma. SLNB should be recommended in patients with T1a melanoma and a high mitotic rate, as even though this combination is uncommon, it is associated with a high risk of SLN metastasis. Sex, acral lentiginous melanoma, and Clark level were also independently associated with survival in our cohort and should be taken into account when devising new staging systems. More studies, however, are needed to validate our findings.

## CONFLICT OF INTEREST

None declared.

## IRB APPROVAL STATUS

The study was approved by the institutional Review Board at Hospital Universitario Reina Sofía, Córdoba (Spain). Acta no 266, ref. 3569.
